# Extracellular-Vesicle-Based Cancer Panels Diagnose Glioblastomas with High Sensitivity and Specificity

**DOI:** 10.3390/cancers15153782

**Published:** 2023-07-26

**Authors:** Melike Mut, Zelal Adiguzel, Canan Cakir-Aktas, Şahin Hanalioğlu, Gamze Gungor-Topcu, Ezgi Kiyga, Ilkay Isikay, Aydan Sarac, Figen Soylemezoglu, Thomas Strobel, Elisabet Ampudia-Mesias, Charles Cameron, Tulay Aslan, Eray Tekirdas, Mutlu Hayran, Kader Karli Oguz, Christine Henzler, Nurten Saydam, Okay Saydam

**Affiliations:** 1 Department of Neurosurgery, Faculty of Medicine, Hacettepe University, Ankara 06230, Turkey; hanalioglu@hacettepe.edu.tr (Ş.H.); isikay@hacettepe.edu.tr (I.I.); tulayaslan@hacettepe.edu.tr (T.A.); eray.tekirdas@hacet.epe.edu.tr (E.T.); 2 Institute of Neurological Sciences and Psychiatry, Hacettepe University, Ankara 06230, Turkey; c.cakiraktas@hacettepe.edu.tr; 3 TUBİTAK, GEBI, Gebze, Kocaeli 41470, Turkey; zeadiguzel@ku.edu.tr (Z.A.); g.gungor@gtu.edu.tr (G.G.-T.); ezgi.kiyga@ogr.iu.edu.tr (E.K.); aydansarac@gmail.com (A.S.); 4 Faculty of Medicine KUTTAM, Koç University, Davutpaşa Street No. 4 Topkapi, Istanbul 34010, Turkey; 5 Department of Pathology, Faculty of Medicine, Hacettepe University, Ankara 06230, Turkey; fsoyleme@hacet.epe.edu.tr; 6 Division of Neuropathology and Neurochemistry, Department of Neurology, Medical University of Vienna, 1090 Vienna, Austria; thomas.stroebel@meduniwien.ac.at; 7 Division of Hematology and Oncology, Department of Pediatrics, Medical School, University of Minnesota, Minneapolis, MN 55455, USA; ampud001@umn.edu (E.A.-M.); camer308@umn.edu (C.C.); 8 Department of Preventive Oncology, Hacettepe University Cancer Institute, Ankara 06230, Turkey; mhayran@hacet.epe.edu.tr; 9 Department of Radiology, Faculty of Medicine, Hacettepe University, Ankara 06230, Turkey; kkoguzhatice@ucdavis.edu; 10 Minnesota Supercomputing Institute, University of Minnesota, Minneapolis, MN 55455, USA; chenzler@umn.edu; 11 ExoMed Diagnostic, Minneapolis, MN 55404, USA; nurtensaydam@yahoo.com

**Keywords:** glioblastoma, extracellular vesicles, biomarkers

## Abstract

**Simple Summary:**

In this study, we used the RNA sequencing of serum EVs isolated from a large cohort of IDH-wt glioblastoma patients and cancer-free healthy controls to uncover new biological tumor markers with prognostic and diagnostic utility. To our knowledge, this is the first study showing that serum-EV-based biomarker panels can be used to predict/diagnose tumor tissue status in terms of IDH1 mutation, MGMT promoter methylation, TERT promoter mutation, and p53 mutation with a high sensitivity and specificity for glioblastoma. These cancer biomarkers are next-generation precision oncology tools that can be used to (i) predict cancer risk at early stages of the disease, which might offer patients more options and a better chance at overcoming these deadly tumors through surgery or other treatment approaches, and (ii) improve patient stratification and treatment regimes.

**Abstract:**

Glioblastoma is one of the most devastating neoplasms of the central nervous system. This study focused on the development of serum extracellular vesicle (EV)-based glioblastoma tumor marker panels that can be used in a clinic to diagnose glioblastomas and to monitor tumor burden, progression, and regression in response to treatment. RNA sequencing studies were performed using RNA isolated from serum EVs from both patients (*n* = 85) and control donors (*n* = 31). RNA sequencing results for preoperative glioblastoma EVs compared to control EVs revealed 569 differentially expressed genes (DEGs, 2XFC, FDR < 0.05). By using these DEGs, we developed serum-EV-based biomarker panels for the following glioblastomas: wild-type IDH1 (96% sensitivity/80% specificity), MGMT promoter methylation (91% sensitivity/73% specificity), p53 gene mutation (100% sensitivity/89% specificity), and TERT promoter mutation (89% sensitivity/100% specificity). This is the first study showing that serum-EV-based biomarker panels can be used to diagnose glioblastomas with a high sensitivity and specificity.

## 1. Introduction

Glioblastoma, which includes grade 4 tumors of the central nervous system (CNS) according to the World Health Organization (WHO), is the most common malignant primary brain tumor in adults, with a median overall survival of only 16–18 months after the diagnosis [1]. Glioblastomas comprise a highly malignant group of tumors that commonly occur in elderly patients (median age at diagnosis, 65 years). Historically, the histopathological diagnosis of glioblastoma was primarily based on the presence of necrosis and/or microvascular proliferation in addition to anaplastic features such as prominent cellular and nuclear atypia, frequent mitotic figures, areas of necrosis, and vascular proliferation. However, in the most recent WHO CNS 2021 classification, the presence of epidermal growth factor receptor (EGFR) amplification and/or whole chromosome 7 gain and whole chromosome 10 loss (+7/−10) and/or telomerase reverse transcriptase (TERT) promoter mutations indicates a molecular diagnosis of glioblastoma, WHO CNS grade 4, in isocitrate dehydrogenase (IDH)-wt astrocytic gliomas [2,3].

The WHO 2016 classification of CNS tumors defines specific glioma entities based on a molecular signature to create optimized prognosis and treatment stratification categories. According to this classification, the most critical molecular signature for these optimized categories for the prognosis and treatment stratification of diffuse gliomas is IDH mutation, and IDH-wt gliomas have a worse prognosis than IDH-mutant gliomas [4,5,6]. In addition to these genetic changes, p53 mutation is the most frequent and earliest detectable genetic alteration and is already present in 60% of precursor low-grade astrocytomas; moreover, the accumulation of other mutations leads to the malignant progression of glioblastoma over time [7].

The clinical management of glioblastoma suffers from a lack of early diagnostic tests. These tumors are mainly diagnosed through the resection/biopsy of the tumor followed by neuroimaging techniques (e.g., magnetic resonance imaging—MRI). Although surgery is the gold standard for diagnosis, this approach only provides a temporal and spatial snapshot of these heterogeneous tumors that displays longitudinal changes due to the selective pressures of ongoing therapy. Furthermore, treatment efficacy is difficult to evaluate in many cases, particularly during early therapy, because therapy-associated tissue inflammation often resembles the effects of disease progression using MRI (termed pseudoprogression) [8]. In addition to pseudoprogression, radionecrosis, tumor progression, and pseudoresponse can be challenging to differentiate through MRI or positron emission tomography (PET) scans [9]. As a result, determining the actual progression of the disease is difficult, which impacts a timely response to treatment failure in patients. This issue has been a challenge in clinical trials, as no reliable surrogate marker is available for disease monitoring. Thus, a noninvasive, longitudinal strategy would be instrumental for many purposes, such as the diagnosis, prognostic assessment, prediction of treatment response, and assessment of tumor progression, as tissue-based tumor profiles are subject to sampling bias, providing only a snapshot of tumor heterogeneity, and cannot be obtained repeatedly. These limitations indicate an unmet need for noninvasive, practical, and flexible approaches for the clinical monitoring of glioblastoma. Circulating biomarkers are an appealing potential solution [10]. Therefore, in this study, we focused on developing patient-serum-derived extracellular vesicle (EV)–glioblastoma tumor marker panels that can be implemented in a clinic to diagnose glioblastoma and to monitor tumor burden and progression as well as tumor response to therapy.

EVs are membrane-bound nanovesicles consisting of small EVs (<200 nm) and medium/large EVs (>200 nm) that are actively released by both healthy cells and cancer cells. EVs are present in biological fluids and are involved in multiple physiological and pathological processes. EVs consist of a lipid bilayer that contains both transmembrane and nonmembrane proteins and miRNAs, mRNAs, and either single- or double-stranded DNA. After docking onto a cell, they deliver cargo and thereby alter molecular activities in the recipient cells. EVs play a critical role in intercellular communication between distant cells and participate in several pathological processes of tumor cells, including proliferation, migration, invasion, and angiogenesis [11,12,13]. The accessibility of EVs in biofluids, such as cerebrospinal fluid (CSF) and blood, provides new diagnostic opportunities for minimally invasive biomarker discovery for glioblastoma. Recent studies have used the miRNA and mRNA content of EVs as a potential diagnostic tool that can also be used in genetic subtyping [14,15,16,17,18,19,20,21]. EVs from glioma patients have been shown to be a source for the detection of clinically relevant prognostic biomarkers, such as IDH1-R132H and EGFRvIII, and have been successfully extracted from blood and CSF [14,21,22,23,24]. Additionally, a higher EV concentration in the plasma of glioblastoma patients than in healthy individuals was demonstrated and linked to tumor recurrence after resection [21]. Overall, EVs are a potential biomarker to diagnose glioblastoma, monitor disease progression, and distinguish patients with tumors from both healthy controls and patients harboring other brain lesions [20,25,26].

In this study, we applied the RNA sequencing of serum EVs isolated from a large cohort of glioblastoma patients and age-matched cancer-free healthy controls to discover new tumor markers with diagnostic and monitoring utility. We used the obtained data to develop cancer panels that can distinguish patients with IDH-wt glioblastoma from controls and predict TERT promoter mutation, MGMT methylation status, and p53 mutation with a high sensitivity and specificity.

## 2. Materials and Methods

### 2.1. Sample Collection

The Institutional Review Board of Hacettepe University Faculty of Medicine approved the study (Ethics number: 14/292-25). All participants provided written informed consent before participating in the study. The study cohort consisted of 91 glioblastoma patients (52 males, 39 females) and 31 healthy, age- and sex-matched control subjects (14 males, 17 females). Preoperative blood samples were collected in nonadditive tubes at the Department of Neurosurgery of Hacettepe University and deidentified using the Neuro-oncology Tumor Repository. Blood samples were allowed to stand at RT for 60 min and centrifuged at 1100× *g* for 15 min at 4 °C. Serum samples were aliquoted into multiple tubes and stored at −80 °C.

### 2.2. Isolation and Characterization of EVs

#### 2.2.1. EV and RNA Isolation

After obtaining serum, 1 mL of serum was exposed to RNase (6.25 µg/mL, Thermo Scientific, Waltham, MA, USA, 12,091,039) for 30 min at RT and centrifuged at +4 °C at 16,000× *g* for 10 min. Supernatants were collected and loaded onto columns. TRIzol was added directly to the columns, and EVs were eluted with Buffer XE. RNA from serum samples was isolated with an exoRNeasy Serum/Plasma Midi Kit (Qiagen, Germantown, MD, USA, 77,164) according to the manufacturer’s instructions. RNA concentration determination and quality checks were performed with small RNA and Pico RNA kits. Serum (0.5, 1, and 2 mL) was used for optimization. Quantitation prior to RNA sequencing was performed with an Agilent RNA 6000 Pico Kit.

#### 2.2.2. Nanoparticle Tracking Analysis (NTA)

EV samples were diluted with PBS at appropriate ratios and measured with the NanoSight NS300 device (NS300, Malvern, UK) [27]. The flow-cell top plate chamber temperature was 25 °C. The camera level was adjusted with video recording to minimize background noise, resulting in an image with a sufficient contrast to identify particles. For each sample, five different 30 s videos were prepared.

### 2.3. Western Blotting

EVs were lysed in a 10X RIPA buffer (Cell Signaling, Cat. No. 9806, Danvers, MA, USA), and a bicinchoninic acid (BCA) assay was used to determine protein concentrations. Protein samples (50 µg) were separated on 10% sodium dodecyl sulfate–polyacrylamide gels and transferred to PVDF membranes. The membranes were blocked with 5% skim milk for 2 h and incubated with primary CD81 (1:1000) (Novus, St. Charles, MO, USA, Cat. No. DB100-65805), TSG101 (1:1000) (Novus, Cat. No. NB200-112), ALIX (1:200) (Santa Cruz, Cat. No. sc-53540), and calnexin (1:1000) (Santa Cruz, sc-46669) antibodies overnight at 4 °C. After washing the unbound primary antibody with 1X TBS-0.1% Tween-20, the membranes were incubated with a secondary antibody (1:100) (Novus, Cat. No. NB7511) for 2 h at RT. An ECL Plus Western blotting Detection System (Cat. No. 32106, Thermo Fisher Scientific, Waltham, MA, USA) was used for visualization. Whole Western blots were added into Supplemental Material S1.

### 2.4. Enzyme-Linked Immunosorbent Assay (ELISA)

EV isolates were diluted 1:10, and a PS Capture Exosome ELISA kit (Anti-mouse IgG POD, #297-79201, Fujifilm, Kanagawa, Japan) was used to quantify CD63 expression. The CD63 protein concentration (ng/mL) in EV isolates was calculated from a standard curve.

### 2.5. cDNA Synthesis

We used a High-Capacity cDNA Reverse Transcription Kit (Applied Biosystems, Beverly, MA, USA) to synthesize cDNA from 1000 ng of total RNA. The conditions for reverse transcription were as follows: step 1, 10 min at 25 °C; step 2, 120 min at 37 °C; and step 3, 5 min at 85 °C, followed by a 4 °C hold. The reaction mixture was mixed with an equal amount of RNA (10 µL).

### 2.6. DNA Preparation

Genomic DNA was isolated from FFPE material using the QIAamp DNA FFPE Tissue Kit (Qiagen) according to the manufacturer’s instructions.

### 2.7. Pyrosequencing

The primers used in genotyping experiments are detailed in Supplementary Table S3.

### 2.8. IDH1 and IDH2

Portions of the IDH1 and IDH2 genes were amplified, spanning mutation sites in codon 132 (IDH1) and codon 172 (IDH2), respectively. Thermal cycling consisted of 45 cycles of denaturing (95 °C, 30 s), annealing (53 °C, 30 s), and elongation (72 °C, 30 s) steps, preceded by an initial denaturation step (95 °C, 15 min) and followed by a final elongation step (72 °C, 6 min). A pyrosequencing analysis was performed with a PyroMark Q24 Qiagen system. Briefly, single-stranded DNA was prepared from 20 μL of a biotinylated PCR product with streptavidin-coated Sepharose beads (GE Healthcare, Chicago, IL, USA) and 0.4 mM of sequencing primers using the PyroMark Vacuum Prep Tool (Qiagen, Hilden, Germany) according to the manufacturer’s instructions.

### 2.9. BRAF and H3F3A

Portions of the BRAF and H3F3A genes were amplified, spanning mutation sites in codon 600 (BRAF) and codons 27 and 34 (H3F3A), respectively. Thermal cycling consisted of 45 cycles of denaturing (94 °C, 30 s), annealing (60 °C, 45 s), and elongation (72 °C, 30 s) steps, preceded by an initial denaturation step (95 °C, 15 min) and followed by a final elongation step (72 °C, 6 min). A pyrosequencing analysis was performed using a PyroMark Q24 Qiagen system as described above with 0.4 mM of sequencing primers.

### 2.10. TERT

A portion of the TERT promoter region was amplified, spanning nucleotide positions −228 and −250. Thermal cycling consisted of 50 cycles of denaturing (94 °C, 30 s), annealing (60 °C, 30 s), and elongation (72 °C, 30 s) steps, preceded by an initial denaturation step (95 °C, 15 min) and followed by a final elongation step (72 °C, 10 min). A pyrosequencing analysis was performed using a PyroMark Q24 Qiagen system as described above with 0.4 mM of sequencing primers.

### 2.11. MGMT

An EpiTect Bisulfite Kit (Qiagen, Cat. No. 59104) was used for the bisulfite treatment of genomic DNA. To determine MGMT promoter methylation status, 50 ng of bisulfite-treated genomic DNA was analyzed using the Therascreen MGMT Pyro Kit (QIAGEN, Cat. No. 971061) according to the manufacturer’s instructions.

### 2.12. RNA Extraction from Serum EVs

One milliliter of the serum sample was treated with Rnase (6.25 µg/mL, Thermo Fisher Scientific, Cat. No. EN0601) for 30 min at RT and centrifuged at +4 °C at 16,000× *g* for 10 min. Supernatants were collected and loaded onto spin columns. TRIzol was added directly to the columns, and EVs were eluted in Buffer XE (Qiagen, Cat. No. 76214). The exoRNeasy Serum/Plasma Midi Kit (Qiagen, Cat. No. 77144) was used to isolate RNA from serum EVs.

### 2.13. Total RNA Sequencing

Before library preparation, Agilent Small RNA (Cat. No. 5067-1548) or RNA 6000 Pico (Cat. No. 5067-1513) kits were used to assess the quality of the RNA samples. A SMART-Seq Stranded Kit (Cat. No. 634444) was used to generate cDNA libraries from 2.5 ng of total RNA. To avoid a loss of the limited amount of starting material, ribosomal RNA (rRNA) depletion was performed before the final PCR amplification step. A High Sensitivity DNA Kit (Agilent Technologies, Santa Clara, CA, USA) was used to verify the size distribution of the sequencing-ready libraries. The cDNA libraries were quantified with the Qubit High Sensitivity DNA kit (Invitrogen, Thermo Fisher Scientific, USA). Equal amounts of indexed 300–400 bp libraries were pooled and paired-end sequenced with HiSeq 2500 and NovaSeq 6000 sequencers (Illumina, San Diego, CA, USA).

### 2.14. Analysis of RNA-seq Data

An RNA sequence data analysis, an ROC curve preparation, and LASSO graphs are presented in Supplementary Material S1. The raw data and the processed gene count tables are now available through the GEO database (GEO-GSE22851).

## 3. Results

### 3.1. Demographic and Clinical Features of the Study Cohort and Transcriptome Analyses of EVs Derived from Glioblastoma Patient Serum

We designed a study comprising a cohort of 91 glioblastoma patients (52 male, 39 female) and 31 control subjects (14 male, 17 female). All subjects in this cohort were glioblastoma patients who were newly diagnosed and received no prior treatment or surgery. All patients were given preoperative dexamethasone (4 mg QID) and antiepileptic agents (levetiracetam, 500 mg BID). Age- and sex-matched subjects in the healthy control group were free of any malignancies or systemic diseases. Blood samples were collected after overnight fasting, and sera were isolated and stored at −80 °C according to the Institutional Review Board protocols approved by the Hacettepe University Faculty of Medicine. The demographic and clinical features of the patients are summarized in Table 1. We next analyzed the status of IDH1, IDH2, p53, BRAF, and H3F3A mutations, MGMT promoter methylation, and TERT promoter C228T mutation for the tumor specimens, as described in the Materials and Methods. Portions of the IDH1 and IDH2 genes were amplified, spanning mutation sites in codon 132 (IDH1) and codon 172 (IDH2), respectively. We found that 85 of 91 patients were IDH1-wt; the IDH1 R132H mutation was found in only 6 patients. These six IDH1-mutated samples from Table 1 were excluded from this study. We did not detect IDH2 mutations in our samples. We also performed p53 IHC staining of our samples and found p53 mutation in 36 of 91 samples (≥50% nuclear staining was considered to indicate mutated p53). MGMT promoter methylation was observed in 42 of 79 patients, and TERT promoter mutation was observed in 57 of 79 patients. ATRX mutation was detected in 4 of 42 patients. We did not detect mutations in BRAF or H3F3A in our cohort (Table 1).

### 3.2. RNA Sequencing of Circulating EVs

**Figure 1:**
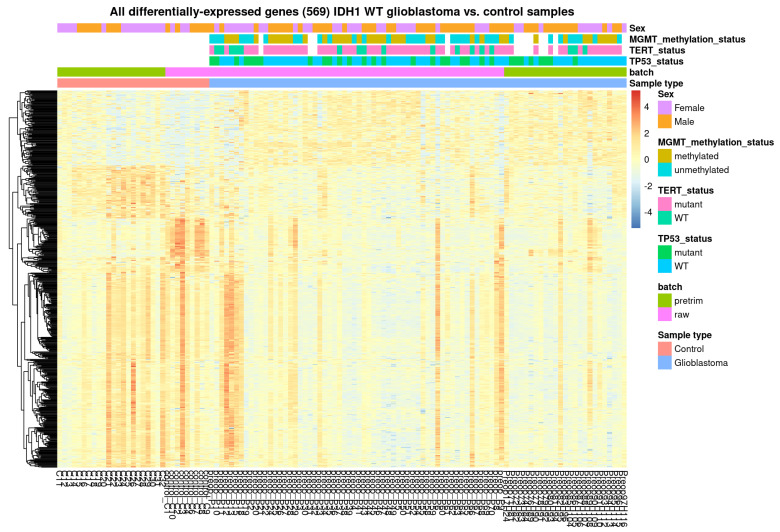
EV heatmap of glioblastomas compared to control EVs. Comparison of preoperative glioblastoma serum EVs (*n* = 85) and control samples (31) revealed 569 differentially expressed genes (DEGs) with an absolute fold-change of 2 and an FDR-adjusted *p* value less than 0.05 (2XFC, FDR < 0.05).

To identify biological markers that can be used in minimally invasive approaches to diagnose and monitor glioblastoma, we used a membrane-based affinity binding protocol to isolate EVs from serum samples of 85 glioblastoma IDH1-wt patients and 31 age- and sex-matched control subjects and performed total RNA-seq on EVs. EVs were characterized by a nanoparticle tracking analysis (NTA) and Western blotting using antibodies against three EV-specific markers, CD63, ALIX, CD81, and calnexin, as negative controls (Supplementary Figure S1), according to the 2018 International Society for Extracellular Vesicles (ISEV) guidelines [28]. Representative results from the NTA analysis of the samples are shown in Supplementary Figure S2. We first isolated EVs from patient serum and compared them to EVs from control subjects and found a significant increase in protein levels of the EV marker CD63 in glioblastoma patient sera compared to control sera. The analysis of preoperative glioblastoma and control samples revealed 569 differentially expressed genes (DEGs), with an absolute fold-change of 2 and an FDR-adjusted *p* value of less than 0.05 (Supplementary Table S1). A heatmap of the 569 DEGs (protein-coding and noncoding) is shown in Figure 1. Overall, 309 of the 569 DEGs are protein-coding. 

### 3.3. Serum-Derived EVs from Patients with IDH1-wt Glioblastoma Have Distinct Transcriptomic Features

**Figure 2:**
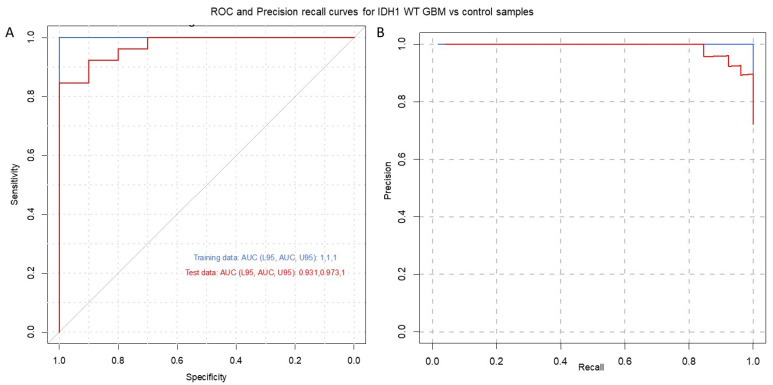
Glioblastoma IDH-wt cancer panel. (**A**) Receiver operating characteristic (ROC) and (**B**) precision–recall curves are shown for IDH1 wild-type vs. control samples. A LASSO-penalized binomial regression model was used, employing 24 of 569 differentially expressed genes as predictors, resulting in 96% sensitivity and 80% specificity for the detection of IDH1 wild-type glioblastomas in comparison to control samples. Training data (blue line) and test data (red line) are shown. Dotted black lines represent the performance of models generated from 100 random permutations of the response variable.

**Figure 3:**
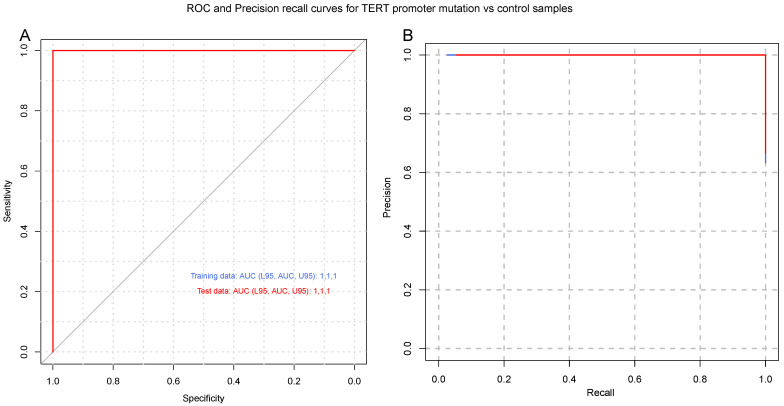
Glioblastoma TERT promoter mutation cancer panel. (**A**) Receiver operating characteristic (ROC) and (**B**) precision–recall curves are shown for the prediction of TERT promoter mutation status. A LASSO-penalized binomial regression model was used, employing 20 of 569 differentially expressed genes as predictors, which resulted in 89% sensitivity and 100% specificity for predicting TERT promoter mutation. Training data (blue line) and test data (red line) are shown. Dotted black lines represent the performance of models generated from 100 random permutations of the response variable.

**Figure 4:**
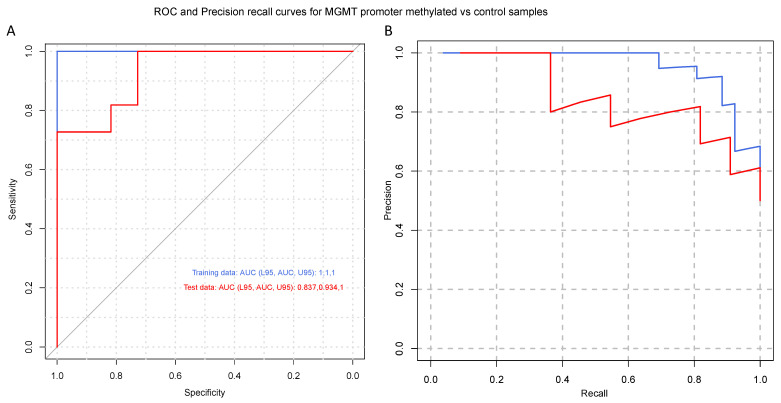
Glioblastoma MGMT promoter methylated cancer panel. (**A**) Receiver operating characteristic (ROC) and (**B**) precision–recall curves are shown for the prediction of MGMT promoter methylation status. A LASSO-penalized binomial regression model was used, employing 17 of 569 differentially expressed genes as predictors, which resulted in 91% sensitivity and 73% specificity for predicting MGMT methylation status. Training data (blue line) and test data (red line) are shown. Dotted black lines represent the performance of models generated from 100 random permutations of the response variable.

**Figure 5:**
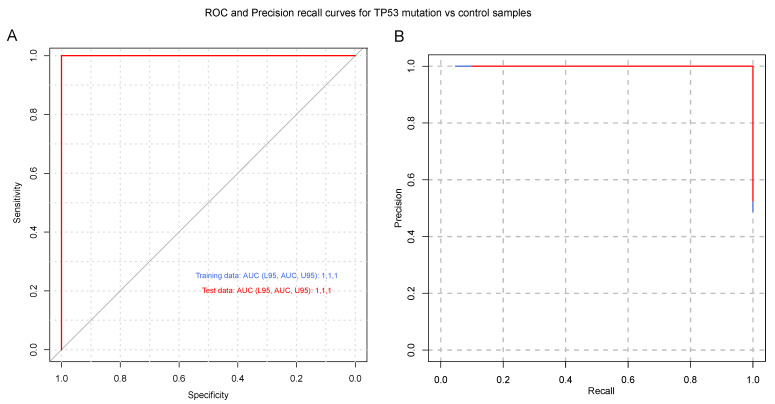
Glioblastoma TP53 mutation cancer panel. (**A**) Receiver operating characteristic (ROC) curve and (**B**) precision–recall curve are shown for the prediction of TP53 mutation status. A LASSO-penalized binomial regression model was used, employing 15 of 569 differentially expressed genes as predictors, which resulted in 100% sensitivity and 89% specificity for predicting TP53 mutation status. Training data (blue line) and test data (red line) are shown. Dotted black lines represent the performance of models generated from 100 random permutations of the response variable.

To identify a gene signature to classify control subjects and patients with glioblastoma (i.e., IDH1 wild-type), we used 569 genes differentially expressed between the two groups as predictors of the diagnosis (0 = control, 1 = glioblastoma). We separated the normalized (counts per million) expression data into a training set (70% of samples) and testing set (30% of samples) and used LASSO regression to develop a predictive model. A receiver operating characteristic (ROC) curve analysis of the test data was used to assess the performance of the final model. This analysis resulted in 96% sensitivity and 80% specificity, using 24 of the 569 DEGs (Supplementary Table S2) from IDH-wt glioblastoma samples (Figure 2). Similar regression analyses were also performed for TERT promoter mutation and MGMT promoter methylation status. As illustrated in Figure 3, the LASSO regression to predict TERT promoter mutation status (predicting TERT mutant glioblastoma compared to control samples) revealed 20 predictor genes that together yielded 89% sensitivity and 100% specificity when applied to the test dataset. The MGMT promoter methylation LASSO regression (predicting glioblastomas with methylated MGMT promoters compared to controls) yielded 91% sensitivity and 73% specificity for the test dataset, using 17 of 569 genes (Figure 4) (Supplementary Table S2). Moreover, the p53 gene mutation cancer panel with 15 of 569 genes showed 100% sensitivity and 89% specificity in identifying p53 mutant glioblastomas from control samples when applied to the test dataset (Figure 5).

**Figure 6:**
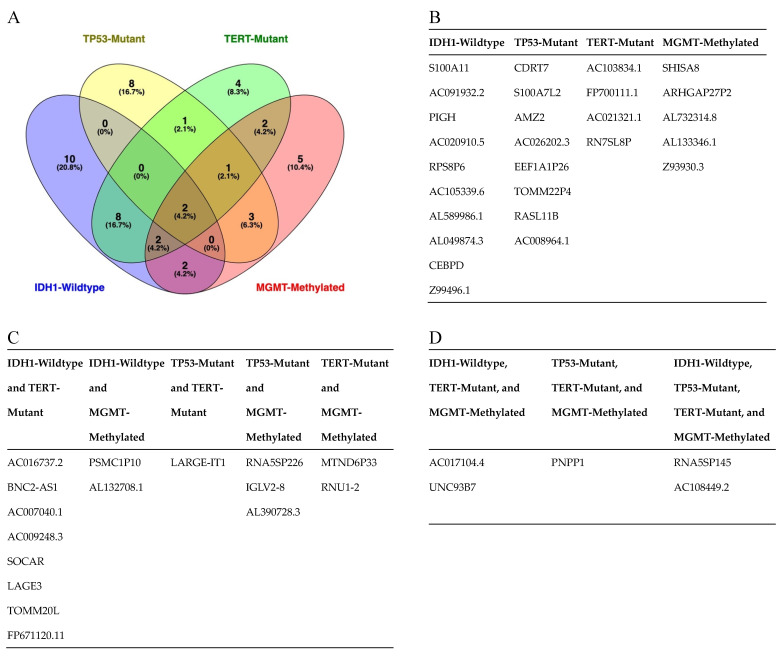
(**A**) Venn diagram of all dysregulated genes (LASSO regression predictors) found in glioblastoma subgroups compared to controls. (**B**) Table of genes dysregulated in one glioblastoma subgroup compared to controls. (**C**) Table of genes dysregulated in two glioblastoma subgroups compared to controls. (**D**) Table of genes dysregulated in three or more glioblastoma subgroups compared to controls.

To compare the dysregulated genes found in each cancer panel, we created a Venn diagram (Figure 6A). From this analysis, we found ten DEGs specific to IDH1-wt glioblastoma, namely, S100A11, AC091932.2, PIGH, AC020910.5, RPS8P6, AC105339.6, AL589986.1, AL049874.3, CEBPD, and Z99496.1. In the TERT promoter mutation cancer panel, we found four DEGs specific to TERT promoter mutational status: AC103834.1, FP700111.1, AC021321.1, and RN7SL8P. We found eight DEGs specific to the p53 gene mutation cancer panel, namely, CDRT7, S100A7L2, AMZ2, AC026202.3, EEF1A1P26, TOMM22P4, RASL11B, and AC008964.1. The MGMT promoter methylation panel contained five DEGs specific to patients with MGMT methylation: SHISA8, ARHGAP27P2, AL732314.8, AL133346.1, and Z93930.3 (Figure 6B). Several DEGs were found in multiple cancer panels. We found two genes, PSMC1P10 and AL132708.1, specific to patients with IDH1-wt and MGMT-methylated glioblastoma. Eight genes, AC016737.2, BNC2-AS1, AC007040.1, AC009248.3, SOCAR, LAGE3, TOMM20L, and FP671120.11, were specific to patients with IDH1-wt and TERT-promoter-mutated glioblastoma. Three genes, RNA5SP226, IGLV2-8, and AL390728.3, were specific to glioblastoma patients with p53 mutations and MGMT methylation. Two genes, MTND6P33 and RNU1-2, were specific to glioblastoma patients with TERT promoter mutations and MGMT methylation. One gene, LARGE-IT1, was specific to glioblastoma patients with mutations in the p53 gene and the TERT promoter (Figure 6C). We found multiple DEGs that were present in three or more cancer panels. Two genes, AC017104.4 and UNC93B7, were found to be specific for patients with IDH1-wt, TERT-promoter-mutated, and MGMT-methylated glioblastoma. There was one gene, PNPP1, specific to glioblastoma patients with MGMT methylation and mutations in the p53 gene and the TERT promoter. Finally, there were two DEGs, RNA5SP145 and AC108449.2, specific to patients with MGMT methylation, p53 mutation, TERT promoter mutation, and IDH1-wt glioblastoma (Figure 6D).

## 4. Discussion

Imaging modalities and tissue biopsies have inherent limitations that render them unsuitable for use in accurate and timely diagnoses and the monitoring of disease progression and treatment response. Tissue biopsies require an invasive procedure and can offer only a snapshot of tumor evolution at a single time point. Imaging modalities are insufficient to distinguish actual tumor progression from treatment artifacts that mimic progression [8,9]; moreover, they require costly instrumentation and time. For brain tumors, detecting serum biomarkers was once challenging due to the blood–brain barrier (BBB), which impedes the release of tumor entities into the bloodstream, even though the integrity of the BBB is compromised in patients with high-grade glioma.

Recently, circulating nucleic acids, tumor cells, and vesicles containing RNA, proteins, and lipids, such as EVs in blood and cerebrospinal fluid, have been under active investigation [29,30,31]. Accordingly, in this study, we focused on developing tumor biomarker panels based on EVs derived from glioblastoma patient serum that can be used at the genetic level in a clinic to diagnose glioblastomas, monitor tumor burden, and assess progression and tumor response to therapy.

Glioblastomas release EVs carrying complex biologically active molecules into the tumor microenvironment, CSF, and bloodstream, and they are thus attractive targets for biomarkers. Obtaining CSF via a lumbar puncture cannot easily be justified as a routine procedure for a glioblastoma diagnosis and follow-up care due to the invasiveness of the procedure and the risk of brain herniation due to the tumor mass effect and other detrimental complications. Obtaining serum is easier because patients with glioblastoma have increased levels of circulating EVs. Tumor-specific EV levels and elevated plasma EV concentrations in glioblastoma patients were found to drop after surgery but rise again at tumor relapse, suggesting that EV dynamics might reflect disease status [21]. Recently, tumor-specific molecules, such as EGFRvIII protein and mRNA and mutant IDH mRNA and DNA, were detected in EVs obtained from glioma cell cultures and blood from glioblastoma patients [14,17,32]. Although these results highlight the diagnostic potential of EVs, the informative value of mutations in a few selected genes affected by recurrent hotspot mutations, such as IDH1 or EGFR, is limited to only a subset of patients harboring these alterations. More comprehensive profiling is necessary to classify tumors with unknown genetic alterations and to monitor changes in the genetic and epigenetic tumor make-up over the course of disease treatment and progression [33].

Reports suggest that exosomal miRNA screening may be used as a predictive biomarker for glioblastoma patients to monitor response to chemotherapy and drug resistance, and recent studies have sought to develop a diagnostic panel to diagnose glioblastoma on the basis of serum samples [34,35]. For example, Manterola et al. found that increased levels of RNU6-1, miRNA-320, and miRNA-574-3p correlated with a glioblastoma diagnosis with a specificity and sensitivity of approximately 86% [26]. By using unbiased high-throughput next-generation sequencing (NGS) and an integrative bioinformatic platform, Ebrahimkhani et al. detected 26 differentially expressed miRNAs in glioblastoma patients compared to healthy controls [20]. They selected a panel of seven miRNAs, miRNA-182, miR-328-3P, miR339-5p, miR340-5p, miR-486-5p, and miR-543, that were able to predict a glioblastoma diagnosis with 91% accuracy. With a multivariate model, four miRNAs, miRNA-182, miR-328-3P, miR340-5p, and miR-486-5p, were able to distinguish glioblastomas from healthy controls with 100% accuracy [20]. Moreover, after using on-chip immunofluorescence to measure the concentrations of the GFAP and TAU proteins in EVs, Lewis et al. reported that it is possible to differentiate the plasma of controls from that of glioblastoma patients, with 60% and 94% accuracy, respectively [36].

In this study, we used the RNA sequencing of serum EVs isolated from a large cohort of IDH-wt glioblastoma patients and healthy controls to uncover new biological markers with prognostic and diagnostic utility. To date, the most crucial molecular marker of tumors of the CNS is IDH1/IDH2 mutations, the identification of which is an essential component of the diagnosis, characterization, and prognosis of diffuse gliomas [37]. In general, an absence of molecular features of glioblastoma should prompt additional molecular testing (e.g., testing for BRAF alterations, testing for histone mutations, and methylome profiling) to achieve a specific diagnosis and to exclude the presence of other IDH-wt gliomas, such as diffuse midline glioma neuroepithelioid tumors, ganglioglioma, pleomorphic xanthoastrocytoma (PXA), and pilocytic astrocytoma [2]. Our study excluded H3K27M- and BRAF V600E-mutated IDH-wt gliomas. Diffuse midline glioma with histone H3K27M mutation, recognized as grade 4 by the WHO in 2016, represents a group of diffusely infiltrating gliomas found in midline CNS structures such as the thalamus, brainstem, and spinal cord [38]. BRAF V600E mutations are more commonly associated with other IDH-wt gliomas, such as circumscribed neuroepithelioid tumors, ganglioglioma, pleomorphic xanthoastrocytoma (PXA), and pilocytic astrocytoma. However, 54% to 93% of epithelioid glioblastomas express BRAF V600E [39]. We describe a gene signature that can distinguish patients with IDH-wt glioblastoma from healthy controls with a high sensitivity and specificity. Indeed, our analysis showed 96% sensitivity and 80% specificity for IDH-wt glioblastoma with a panel of 24 of 569 DEGs (Figure 2). A regression analysis was also performed for MGMT promoter methylation and TERT mutation status. As depicted in Figure 4, the MGMT promoter methylation panel showed 91% sensitivity and 73% specificity for a panel of 17 of 569 genes. As shown in Figure 3, a panel consisting of 20 of 569 genes detected glioblastoma TERT promoter mutations with 89% sensitivity and 100% specificity. Moreover, the p53 gene mutation cancer panel with 15 of 569 genes showed 100% sensitivity and 89% specificity for differentiating p53 mutant glioblastomas from control samples when applied to the test dataset (Figure 5). Hence, our panel is highly sensitive and specific for diagnosing IDH-wt glioblastoma or IDH-wt glioblastoma on the basis of molecular features according to the WHO grade 4 2021 classification.

### 4.1. Potential Significance

Considering the existence of challenging situations in which equivocal neuroimaging studies are unable to indicate a primary diagnosis (for example, differentiating metastatic brain disease, lymphoma, inflammatory conditions, etc.), noninvasive serum biomarkers are of great value. Furthermore, monitoring serum biomarkers can be instrumental in evaluating the response to treatment and, in the differential diagnosis of pseudoprogression versus tumor progression, it can even be instrumental in considering the heterogeneous and genetically evolving nature of glioblastomas. Although the neurosurgeons who performed this study are advocates of redo surgeries, unfortunately, this approach might be limited in some cases due to inherent issues related to patient comorbidities and treatment side effects. In these cases, serum biomarkers have great potential as noninvasive, relatively inexpensive, safe, and repeatable diagnostic and monitoring tools.

### 4.2. Limitations

Our initial aim was to develop a panel for use as a serum-based diagnostic tool to distinguish glioblastoma patients from healthy subjects. Our series of glioblastoma samples represents IDH-wt tumors without BRAF and H3K27 mutations. The glioblastoma IDH1-wt panel represented in Figure 2 needs to be further evaluated in IDH1-wt tumors with BRAF and H3K27 mutations. Additionally, it is likely that the DEGs discovered in this cohort will change in response to disease progression and treatment. Due to a lack of postoperative sample data analysis, it is also possible that some of the DEGs might indicate a response to preoperative surgery treatment. It would be interesting to investigate whether there is a possible correlation between glioblastoma DEGs and lower grades of gliomas.

## 5. Conclusions

To our knowledge, this is the first study showing that serum-EV-based biomarker panels can be used to predict/diagnose tumor tissue status in terms of IDH1 mutation, MGMT promoter methylation, TERT promoter mutation, and p53 mutation with a high sensitivity and specificity for glioblastoma. However, an independent validation cohort is required to investigate whether these cancer panels can be used in a clinic.

This panel was developed using a relatively large cohort with a meticulous follow up and a detailed study of molecular markers predicted before the 2021 WHO classification was released. Although our NGS analyses reflect the heterogeneous and diverse nature of glioblastomas, the panel can efficiently be utilized in the diagnosis of glioblastoma. A validation cohort involving IDH-wt glioblastomas and BRAF-H3K27-mutated IDH-wt gliomas is needed. Furthermore, a prospective study should be carried out in the future to validate the panel for clinical application via the disease monitoring of glioblastoma patients with evaluations of tumor burden and response to therapy.

## Figures and Tables

**Table 1:** Demographic and clinical features of patients.

Controls	Male	Female	Total #	MGMT Status	Male	Female	Total #
Number of patients	14	17	31	Unmethylated	22	15	37
Age, years	51.5 (27–86)	47 (29–61)		Methylated	24	18	42
Median (min.–max.)				NA	6	6	12
**Glioblastoma**				**TERT promoter status C228T**			
Number of patients	52	39	91	Wild-type	11	11	22
Age, years	55.02 (24–84)	54.44 (24–81)		Mutant	35	22	57
Median (min.–max.)				NA	6	6	12
**IDH status (R132H)**				**ATRX status**			
Wild-type	48	37	85	Wild-type	18	20	38
Mutant	4	2	6	Mutant	1	3	4
NA				NA			
**IDH2 status (R172H)**				**BRAF status (600)**			
Wild-type	52	39	79	Wild-type	46	33	79
Mutant	0	0	0	Mutant	0	0	0
TP53 status				H3F3A status (27/34)			
Wild-type	31	24	55	Wild-type	46	33	79
Mutant	21	15	36	Mutant	0	0	0
NA	0	0	0	NA	6	6	12

## Data Availability

The raw data and the processed gene count tables are available through the GEO database under GSE228512.

## Supplementary Materials

The following supporting information can be downloaded at https://www.mdpi.com/article/10.3390/cancers15153782/s1, References [40,41,42,43,44,45,46,47,48,49] are cited in the supplementary materials. Figure S1: EV markers; Figure S2: Representative results from the NTA analysis of the samples; Table S1: The list of differentially expressed genes (DEG); Table S2: The list of DEG in each panel; Table S3: The list of primers used in genotyping experiments.
