# Supplementary material for: Extracellular-Vesicle-Based Cancer Panels Diagnose Glioblastomas with High Sensitivity and Specificity
Source: Cancers (Basel). 2023 Jul 26;15(15):3782. doi: 10.3390/cancers15153782 (PMC10417317; doi:10.3390/cancers15153782)
Supplement: Supplementary file 1 [file cancers-15-03782-s001.zip › Suppl Material 1.pdf]

Supplementary Material:

Reads from the 128 samples that underwent total RNA sequencing were processed using the CHURP pipeline (1). Briefly, read quality was assessed with fastqc (2), and reads were trimmed using trimmomatic (v.33) (3) to remove the first 3 base pairs of R2 (as these are from the SMART-seq Stranded adapter), adapters and low quality bases. Reads were aligned to hg38 using hisat2 (4) and assigned to genes in Ensembl GrCh38 release 100 using Rsubread (5).

In addition to protein-coding RNA, total RNA from exosomal vesicles frequently contains rRNA, repeats, and other ncRNA. To understand how reads were distributed across RNA classes, we used ALFA (v1.11) (6) with the GRCh38 Ensembl release 100 gtf modified to include non-genic repeats from the UCSC Genome Browser repeatmasker track.

Subsequent analyses were carried out in R (v.4.0.4). Prior to differential expression analysis, 12 samples were removed: one sample was from a patient undergoing treatment for athlete's foot, one had incomplete clinical data, four had fewer than 150,000 reads assigned to genes, and six were IDH1 mutants. This yielded a final dataset with 85 glioblastoma patient samples (48 male, 37 female) and 31 control samples (14 male and 17 female). Data were filtered to remove genes less than 100bp in length, and were lowly expressed (filtered using edgeR's filterByExpr with default settings except min.count set to 3) prior to differential expression. Differential expression analysis was carried out using edgeR (7) with sequencer (HiSeq 2500 and NovaSeq 6000) and sex included as factors in the analysis. Differential expression comparisons are outlined in this material. We used LASSO regression, as implemented

by the 'glmnet' package (4, 8) in R (v.4.0.4) (9), to create a logistic regression model with L1 regularization. Data were divided into training (70%) and test (30%) sets, stratified by sequencer batch. To estimate lambda, the tuning parameter for the penalty term for the data, cross-validation (via cv.glmnet) was run 100 times on the training dataset, and the mean squared errors were averaged to identify the lambda with the lowest average MSE across all runs. Using that lambda value, a single, final LASSO regression was run on the training data to create the final model. All datasets used in LASSO regression were unbalanced. To assess the effect of the unbalanced design on the model performance, each LASSO model was regenerated 100 times, randomly permuting the response variable each time. ROC and precision-recall curves were prepared using pROC (10). The raw data and the processed gene count tables are available through the GEO database under GSE228512.

Whole Western blots of the Supplementary Figure S1 is presented as follows:

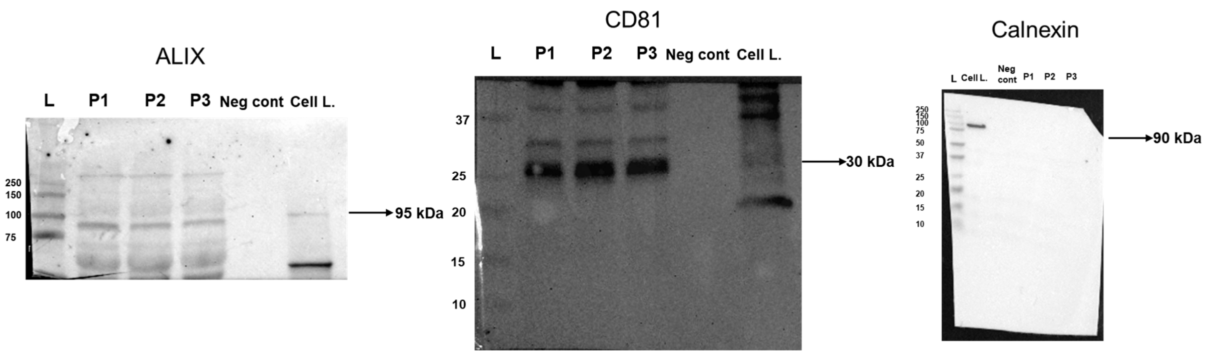

References

1. Baller, J., Kono, T., Herman, A., and Zhang, Y. (2019). CHURP: A Lightweight CLI Framework to Enable Novice Users to Analyze Sequencing Datasets in Parallel. In Proceedings of the Practice and Experience in Advanced Research Computing on Rise of the Machines (learning) (PEARC '19). Association for Computing Machinery, New York, NY, USA, Article 96, 1–5. DOI: https://doi.org/10.1145/3332186.3333156
2. Andrews, S. (2010). FastQC: A Quality Control Tool for High Throughput Sequence Data.
3. Bolger, A., Lohse, M., Usadel, B. (2014). Trimmomatic: a flexible trimmer for Illumina sequence data, *Bioinformatics*, Volume 30, Issue 15, 1 August 2014, Pages 2114–2120. DOI: https://doi.org/10.1093/bioinformatics/btu170
4. Kim, D., Langmead, B. & Salzberg, S. (2015). HISAT: a fast spliced aligner with low memory requirements. *Nat Methods* **12**, 357–360 (2015). https://doi.org/10.1038/nmeth.3317
5. Liao, Y., Smyth, G.K., Shi, W. (2019). The R package Rsubread is easier, faster, cheaper and better for alignment and quantification of RNA sequencing reads. Nucleic Acids Res. 2019 May 7;47(8):e47. DOI: 10.1093/nar/gkz114.
6. Bahin, M., Noël, B.F., Murigneux, V., *et al* (2019). ALFA: annotation landscape for aligned reads. BMC Genomics 20, 250 (2019). DOI: https://doi.org/10.1186/s12864-019-5624-2
7. Robinson, M.D., McCarthy, D.J., Smyth, G.K. (2010). edgeR: a Bioconductor package for differential expression analysis of digital gene expression data. Bioinformatics, 26(1), 139-140. DOI: 10.1093/bioinformatics/btp616.
8. Friedman, J., Hastie, T., Tibshirani, R. (2010). “Regularization Paths for Generalized Linear Models via Coordinate Descent.” Journal of Statistical Software, 33(1), 1–22. DOI:10.18637/jss.v033.i01, https://www.jstatsoft.org/v33/i01/.
9. R Core Team (2022). R: A language and environment for statistical computing. R Foundation for Statistical Computing, Vienna, Austria. URL: https://www.R-project.org/.
10. Robin, X., Turck, N., Hainard, A. *et al* (2011). pROC: an open-source package for R and S+ to analyze and compare ROC curves. *BMC Bioinformatics* **12**, 77 (2011). https://doi.org/10.1186/1471-2105-12-77
