# Supplementary figures and images for: Extracellular-Vesicle-Based Cancer Panels Diagnose Glioblastomas with High Sensitivity and Specificity
Source: Cancers (Basel). 2023 Jul 26;15(15):3782. doi: 10.3390/cancers15153782 (PMC10417317; doi:10.3390/cancers15153782)

## Slide 1
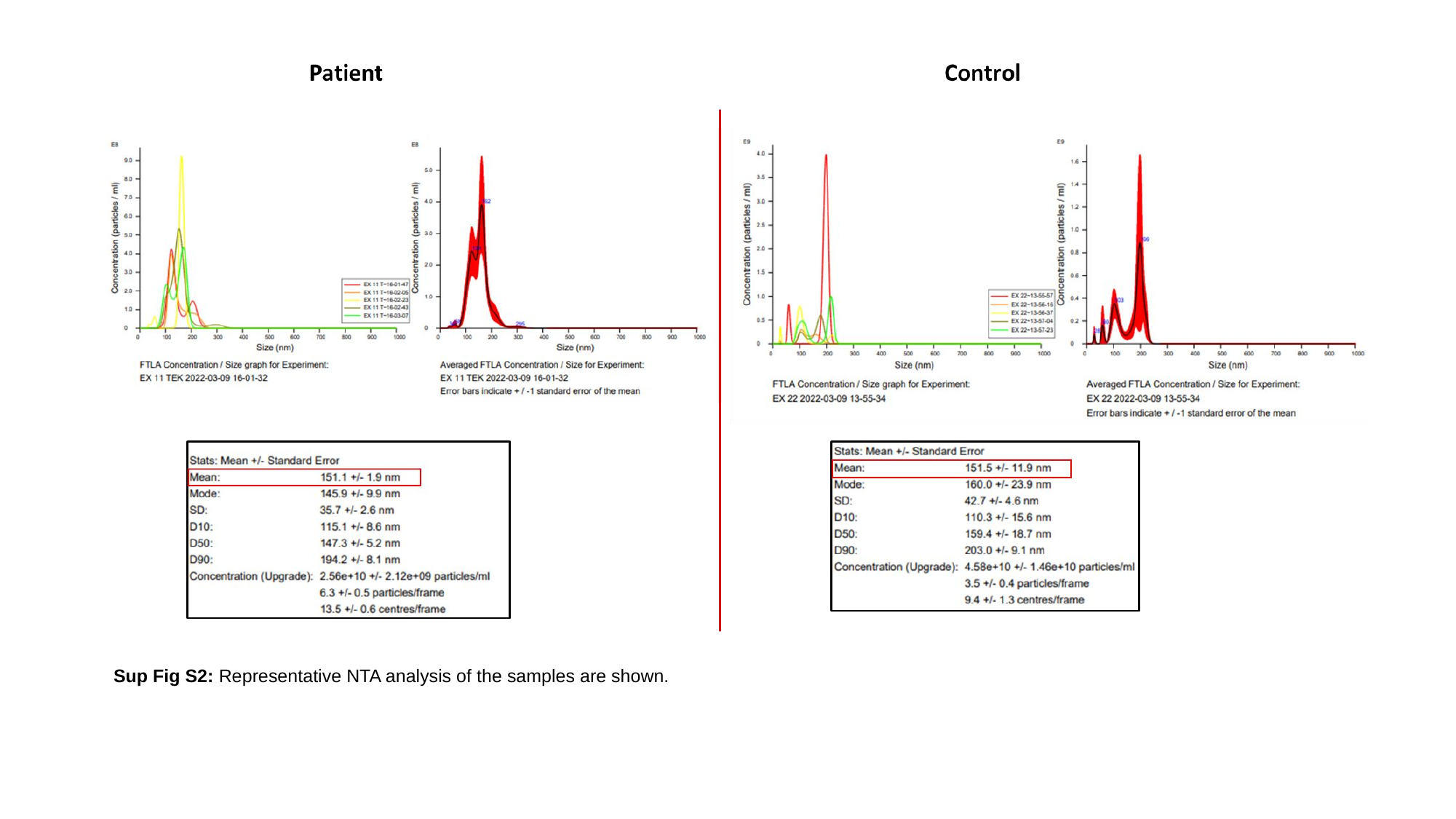

Sup Fig S2: Representative NTA analysis of the samples are shown.

Supplement: Supplementary file 1 [file cancers-15-03782-s001.zip › Supplementary_Figure S2.pptx]
